# Diffraction data analysis in the presence of radiation damage

**DOI:** 10.1107/S0907444909040177

**Published:** 2010-03-24

**Authors:** Dominika Borek, Marcin Cymborowski, Mischa Machius, Wladek Minor, Zbyszek Otwinowski

**Affiliations:** a University of Texas Southwestern Medical Center at Dallas, 5323 Harry Hines Boulevard, Dallas, TX 75390, USA; b University of Virginia, Charlottesville, VA 22908, USA

**Keywords:** radiation-induced specific changes, relative *B* factor, scaling *B* factor, experimental phasing, synchrotron radiation

## Abstract

In macromolecular crystallography, the acquisition of a complete set of diffraction intensities typically involves a high cumulative dose of X-ray radiation. In the process of data acquisition, the irradiated crystal lattice undergoes a broad range of chemical and physical changes. These result in the gradual decay of diffraction intensities, accompanied by changes in the macroscopic organization of crystal lattice order and by localized changes in electron density that, owing to complex radiation chemistry, are specific for a particular macromolecule. The decay of diffraction intensities is a well defined physical process that is fully correctable during scaling and merging analysis and therefore, while limiting the amount of diffraction, it has no other impact on phasing procedures. Specific chemical changes, which are variable even between different crystal forms of the same macromolecule, are more difficult to predict, describe and correct in data. Appearing during the process of data collection, they result in gradual changes in structure factors and therefore have profound consequences in phasing procedures. Examples of various combinations of radiation-induced changes are presented and various considerations pertinent to the determination of the best strategies for handling diffraction data analysis in representative situations are discussed.

## Abstract 2

Radiation-induced decay of crystal diffraction and additional specific chemical changes of macromolecules forming the crystal lattice are currently two of the main limiting factors in the acquisition of macromolecular diffraction data and macromolecular structure determination. Data-processing and phasing protocols are discussed in the context of radiation-induced changes.

## 1. Introduction

With modern X-ray sources, even for cryocooled crystals, the limit of crystal life is reached in times ranging from seconds at the strongest third-generation undulator synchrotron beamlines (http://biosync.rcsb.org/allbeamlines/allbeam.html) to hours at the most intense home sources (Yang *et al.*, 1999 ▶). For this reason, the dose from crystal exposure is now mostly determined by the experimental strategy rather than by X-ray source intensity limitations. Macromolecular crystals inherently scatter weakly, so reaching the highest possible scattering intensity is always desirable in order to minimize random effects (Borek *et al.*, 2003 ▶; Popov & Bourenkov, 2003 ▶; Bourenkov & Popov, 2006 ▶). The decay of half the total diffraction intensity defines the upper limit for X-ray dose (Henderson, 1995 ▶; Owen *et al.*, 2006 ▶; Kmetko *et al.*, 2006 ▶). However, chemical and physical changes induced by X-ray photons during data collection may result in the deterioration of merging statistics, sometimes long before reaching half the intensity decay (Borek *et al.*, 2007 ▶; Zwart *et al.*, 2004 ▶), prompting experimenters to limit the exposure. Even if limiting the exposure preserves less damaged states of the crystal, it often hinders solution of a structure owing to the collected data being either too weak or having insufficient multiplicity of observations to perform experimental phasing (Zwart *et al.*, 2004 ▶). Understanding how radiation-induced specific effects manifest themselves in data is essential to making informed choices about exposure strategy.

These effects can be analyzed from two perspectives, starting with the physics of interactions between X-rays and matter. A good example of such analysis is the program *RADDOSE*, which calculates the X-ray absorption depending on the crystallization conditions, the particular composition of the protein and the X-ray wavelength (Murray *et al.*, 2005 ▶; Paithankar *et al.*, 2009 ▶). The results of this and similar analyses are used as one of the intermediate steps in the process of planning the experiment. We will focus on the other perspective, which starts with analyzing the patterns in X-ray diffraction data. Characteristic data patterns may exist in both real and reciprocal space. The identified patterns may be used at various points of different crystallographic procedures: (i) to plan the next experiment (sometimes even a continuation of the current one; Bourenkov & Popov, 2006 ▶); (ii) to correct data in reciprocal space, *e.g.* to apply a decay correction (Borek *et al.*, 2007 ▶; Otwinowski *et al.*, 2003 ▶) and zero-dose extrapolation (Borek *et al.*, 2007 ▶; Diederichs *et al.*, 2003 ▶; Blake & Phillips, 1962 ▶); and (iii) to combine real-space and reciprocal-space patterns for phasing (Ravelli *et al.*, 2003 ▶; Schiltz & Bricogne, 2007 ▶; Schiltz *et al.*, 2004 ▶) and to infer chemical characteristics of macromolecules forming a particular crystal lattice (Fütterer *et al.*, 2008 ▶; Yano *et al.*, 2005 ▶).

In this article, we describe the main data patterns, how to recognize these, when to expect them and how they have impacted on data collection and phasing in a range of specific cases.

## 2. Sources of the analyzed diffraction data

A data set from a crystal of APC5871, a Midwest Center for Structural Genomics project, was provided by Marianne Cuff, who collected it on beamline 19-ID of the Structural Biology Center (SBC) at the Advanced Photon Source (APS). An APC35880 data set (PDB code 1t0t; M. Gilski, D. Borek, Y. Chen, F. Collart, A. Joachimiak & Z. Otwinowski, unpublished work) was collected from an SeMet-containing crystal on the same beamline. A data set from a crystal of bovine pancreatic trypsin inhibitor (BPTI) corresponding to PDB code 1g6x was provided by Mariusz Jaskolski and was collected on EMBL beamline X11 at DESY as described previously (Addlagatta *et al.*, 2001 ▶). A data set from a crystal of NaI-841 (Borek *et al.*, 2007 ▶), isomorphous with PDB deposition 1r61, was provided by Jana Maderova, who collected it on beamline 19-BM of the SBC at the APS. A data set from a crystal of thiamine-binding lipoprotein p37 from *Mycoplasma hyorhinis*, referred to here as p37n33 (C. Dann, unpublished work), was provided by Charles Dann III, who collected it at Cu *K*α wavelength with an FR-E Super Bright X-ray generator (Rigaku). Data from a crystal of Tp0655 lipoprotein from *Treponema pallidum*, corresponding to PDB code 2v84, were acquired using the same source, as described previously (Machius *et al.*, 2007 ▶). Data from a crystal of proto-oncogene vav, referred to here as VAV, were pro­vided by the Structural Biology Laboratory at University of Texas Southwestern Medical Center at Dallas. Three VAV data sets were collected on beamline 19-ID of the SBC at the APS. The first and second sets were from the same position of the crystal in the beam and the third was obtained by translating the crystal along the rotation axis to expose fresh potentially undamaged parts.

All diffraction data sets used were reprocessed with *HKL*-3000 (Minor *et al.*, 2006 ▶; Otwinowski & Minor, 1997 ▶, 2000 ▶), optimizing scaling and error model parameters (Otwinowski *et al.*, 2003 ▶; Borek *et al.*, 2003 ▶; Table 1 ▶).

## 3. Results and discussion

### 3.1. The impact of X-ray radiation on diffraction intensities

In the discussion, we differentiate between ‘radiation damage’, referring to all X-ray-induced changes in the diffraction data, and the ‘decay of diffraction pattern’, which refers specifically to the resolution-dependent weakening of the diffraction pattern.

During X-ray exposure, owing to the cascade of electronic events resulting from the absorption of X-ray photons, atoms are randomly displaced from their initial positions in the crystal lattice. When the mobility of atoms is restricted by cryocooling, the typical displacements are expected to be on the scale of 1 Å or less. Some of the atoms in the crystal asymmetric unit may be displaced sooner than others. When considering how these effects impact on the diffraction data, the consequences, owing to Fourier transform properties, are very different for the average displacement rate and for departures from it.

The average Gaussian-like local displacement in real space will result, from the Fourier transform property of convolution, in the decay of diffraction intensities, which is resolution-dependent but otherwise globally uniform in reciprocal space. This effect is a consequence, through the Fourier transform, of an averaged value of highly localized properties in real space, so one would expect it to be isotropic, irrespective of whether or not the overall diffraction is isotropic. Additionally, relative rearrangements of the unit cells may also contribute to changes in the diffraction pattern. For uncooled crystals, it is the dominant source of resolution-dependent intensity decay. However, for cryocooled crystals unit-cell changes are typically so minuscule (Borek *et al.*, 2007 ▶; Ravelli *et al.*, 2002 ▶; Murray & Garman, 2002 ▶) that the contribution of lattice disorder to the overall diffraction decay is not significant. In some special cases, discussed in §[Sec sec3.1.3]3.1.3, nonlinear effects may cause lattice disorder, but this will have more consequences than solely the overall decay of the diffraction pattern.

The departures from the average displacement rate are represented by localized changes in electron density, which is calculated using Fourier coefficients already corrected for the radiation decay. From the Fourier theorem, these localized changes in electron density will affect all reflections. As these localized changes accumulate, the decay-corrected intensity of a particular reflection will drift from its initial value, either decreasing or increasing (Blake & Phillips, 1962 ▶). Even if these changes have different directions, they will have a common linearity or a lack of linearity, mirroring the linearity of changes in real space.

#### 3.1.1. Description of diffraction-pattern decay and its role as the proxy of dose

The resolution-dependent decay of diffraction intensities was first noticed a long time ago (Blake & Phillips, 1962 ▶). Ever since, it has been commonly corrected by applying a scaling (relative) *B*-factor correction, which is a specific case of exponential modeling in the scaling of diffraction data (Arnott & Wonacott, 1966 ▶; Otwinowski *et al.*, 2003 ▶; Otwinowski & Minor, 1997 ▶; Smith & Arnott, 1978 ▶). In this approach, scaling factors *B*_rel_ are applied separately to batches of data, *e.g.* consecutive diffraction images, by using the following multiplicative scale factor,
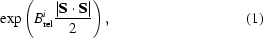
where *B^i^*_rel_ is the scaling (relative) *B* factor for data batch *i* and **S** is the diffraction vector. All the *B*_rel_ are determined together by comparing the intensities of symmetrically equivalent observations measured at different times, *i.e.* at different images and at different doses.

Equation (1) follows from the diffraction intensities *I_hkl_* being reduced by thermal vibrations described by the Debye–Waller factor exp(−2*B*sin^2^/λ^2^), resulting in modification of the measured intensities *I* ^*m*^ *_hkl_*,
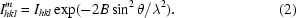
The diffraction-vector magnitude is given by *S* = (2sinθ)/λ, which is equivalent to sinθ/λ = *S*/2, where **S** is a diffraction vector, λ is the wavelength and θ is the Bragg angle. Thus, we can express the measured intensity *I* ^*m*^ *_hkl_* as
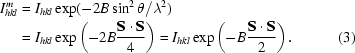
The goal of scaling is to generate a model of the data which is the best description of the measured intensities. Thus, we are interested in calculating how the values of *I_hkl_* change during data collection owing to increasing thermal vibrations or any other diffusive process described by the *B* factor,
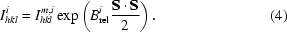
One of the advantages of using the scaling *B* factor compared with other scaling approaches is its ability to estimate the decay of the diffraction pattern even without returning to the starting orientation of the crystal for additional measurements. In the case of anisotropic diffraction, there is no other practical alternative to monitoring a continuously rotating crystal, as the average intensity of diffraction in the image may increase and decrease with rotation owing to anisotropy and not necessarily owing to crystal decay.

As noticed by Kmetko *et al.* (2006 ▶) and also by others (Borek *et al.*, 2007 ▶), accumulating X-ray dose at a particular temperature produces the same scaling *B*-factor increase irrespectively of how the same dose was generated by different combinations of mass-absorption coefficients, exposure time and beam intensity. This relationship between the dose and the *B*_rel_ increase can be explained by the statistical properties of atom displacements generated by X-ray radiation. In cryocooled crystals, we do not expect atoms to move by large distances as a consequence of ionizing radiation; however, small shifts are possible. During the diffraction experiment, each atom in the structure is randomly displaced by many cumulative independent events and if these displacements are individually small the central limit theorem applies. In con­sequence, cumulative displacement is described by a Gaussian distribution, the squared width of which increases linearly with dose, as increasing the dose generates proportionally more individual small displacements. The global scale is not expected to change, since the number of atoms in the X-­ray beam remains the same, and so the decay *B*_rel_ is fully described by a resolution-dependent Gaussian term with a width defined through the factor. Frequently, for many practical reasons, calculation of the dose absorbed by the crystal may be inaccurate or impossible. Even if *B*_rel_ is an indirect measure of dose, it describes a physical property that is directly related to interactions of photons with the crystal, making it a good proxy of the dose.

**Figure 1:**
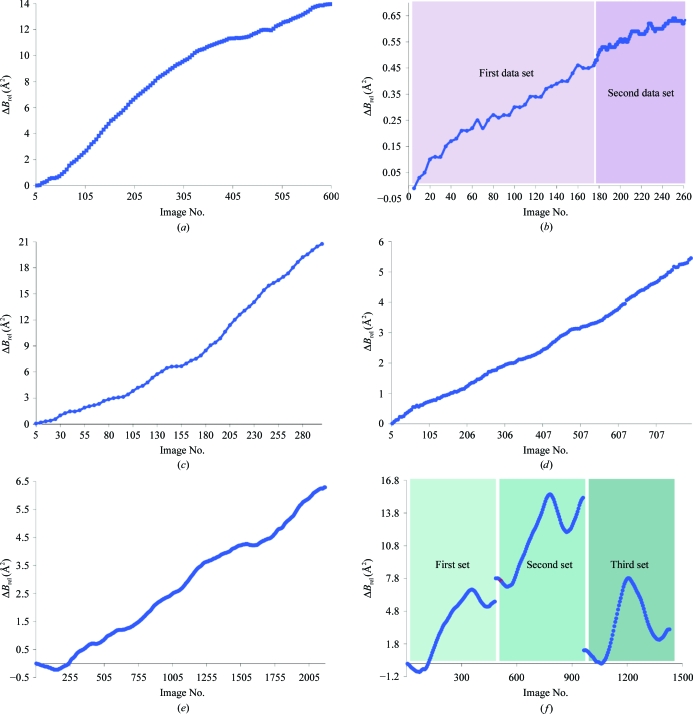
Linear increase of the scaling (relative) *B* factor for various diffraction data sets: (*a*) APC35880, (*b*) BPTI, (*c*) NaI-841, (*d*) p37n33, (*e*) Tp0655, (*f*) VAV. In cases where the crystal was smaller than the beam size (APC35880, BPTI, NaI-841 and p37n33, Tp0655) there is little fluctuation in *B*-factor behavior. VAV represents a case where the crystal was larger than the beam and in which three data sets were collected, with the third data set being acquired after moving the crystal to a new position.

The above reasoning agrees well with experimental observations. For all crystals considered here that were fully and uniformly exposed in the beam (APC35880, BPTI, NAI-841, p37n33 and Tp0655) the scaling *B* factor increases linearly with time (Fig. 1 ▶). If the crystal, for example owing to its shape, as in the case of the VAV crystal, is not fully bathed in the X-­ray beam, then the overall decay will be the weighted average of the decay of different parts of the crystal that are exposed to different doses (Fig. 1 ▶). Imperfect centering may introduce a more complex geometrical dependence of the scaling *B* factor on data-collection time. The VAV data set also shows that *B*_rel_ can be used to make decisions on when to translate the crystal to a new position. The strategy of translating the crystal to expose fresh parts is often employed in the case of crystals with an elongated shape. Of the three VAV data sets, the first two were collected at the same position but with ϕ = 0° for the first data set and ϕ = 180° for the second data set, whereas the third data set was collected at a different crystal position. The scaling *B*-factor increase shows not only that the crystal was larger than the X-ray beam but also that in the case of the first and second data sets the same parts of the crystal were exposed. The scaling *B*-factor behavior for the third data set is almost identical to that of the first data set, showing that fresh sections of the crystal were indeed exposed. It is important to notice that even for diffraction data sets collected at a home source (p37n33 and Tp0655) a significant scaling *B*-factor increase is observed over the 2–3 d of data collection, which is consistent with the known intensity of one of the strongest rotating-anode sources.

#### 3.1.2. Specific radiation-induced changes

Localized in real space, the specific changes induced by X-ray radiation are a complex function of radiation chemistry. Each absorbed photon generates hundreds of secondary ionization events at distances much larger than the unit-cell repeat in the crystal, so effectively the changes arising from these secondary events (O’Neill *et al.*, 2002 ▶) as well as those arising from the recombination of their products are not localized at the absorption site. Localized changes can be identified by calculating the corresponding electron-density map, which represents the difference between the initial and radiation-exposed states.

Our calculation of a radiation-damage difference electron-density map (RDDEM) starts with fitting a linear function of diffraction intensity with dose to observations already corrected for decay (§[Sec sec3.1.1]3.1.1), using an independent fit for every unique diffraction index. The slope coefficients of the fitted line, by definition, represent the rate of change in structure factor squared with respect to dose. In the case of there being a limited number of observations per unique *hkl* the problem of extracting components of the signal simultaneously can be ill-posed, *i.e.* it may lead to a highly inaccurate solution. The inherent lack of knowledge in this situation has a detrimental effect on further stages of analysis, possibly preventing solution of the phase problem. The chosen method to address this issue is to calculate the maximum *a posteriori* estimate (MAP) based on the observation that the expected magnitude of a normalized signal is unity. In the case of a one-component signal, *e.g.* only Bijvoet differences, this is equivalent to a Wiener filter. In crystallography, the Wiener filter-equivalent approach has been previously discussed by Giacovazzo *et al.* (2001 ▶). In the case of multiple signals, *e.g.* Bijvoet differences considered together with non-isomorphisms, the MAP approach is equivalent to Tikhonov regularization (Tikhonov & Arsenin, 1977 ▶) with unity as the regularization constant. The Tikhonov regularization is applied during a determination of the components of the signal to the least-squares matrix **A** = **UΣV** ^**T**^, with singular values *a_i_* to generate the solution 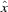 = **UDV^T^b**, where **D** has diagonal values *D_ii_* = *a_i_*(*a_i_* ^2^ + α^2^), with α representing the regularization constant. In our case α is unity, since all signal components are normalized. This signal normalization means that the expected value (r.m.s.) for a particular source of signal, *e.g.* anomalous differences, radiation damage *etc.*, is also unity. Such use of Tikhonov regularization has a clear Bayesian meaning derived from a generalization of the Wiener filter idea to the case of multiple signals.

The results of this procedure, once we have determined the phases, can be displayed in real space as the radiation-damage rate map, which is the Fourier transform of the slope coefficients in the linear fit. This electron-density map is a difference map between the zero radiation dose and any accumulated dose at which the assumption of linearity is still valid. Any non-uniformity of exposure or even a lack of absolute dose calibration does not affect the result. The magnitude (r.m.s.) of radiation-induced specific changes in real space is equivalent by Parseval’s theorem to the sum of the squared difference map coefficients. Therefore, it is convenient to use the index *R*_R_ = 〈Δ*F* ^2^〉^1/2^/〈*F* ^2^〉^1/2^ (Borek *et al.*, 2007 ▶), which expresses the magnitude of specific radiation changes as a fraction of the native signal.

The *R*_R_ index is an approximately linear function of the X-­ray dose in a particular experiment. Thus, to compare the rates of radiation-induced specific changes in different experiments it has to be normalized, which we accomplish by calculating *R*_R_/Δ*B*_rel_ using the scaling *B* factor as a proxy of the dose. There is clearly large variability in the *R*_R_/Δ*B*_rel_ ratio between different proteins (Table 2 ▶). The NaI-841 crystal shows a relatively slow rate of radiation-induced specific changes, *R*_R_/Δ*B*_rel_ = 0.32% Å^−2^, possibly owing to the pre­sence of 0.5 *M* sodium iodide in the cryoconditions (Borek *et al.*, 2007 ▶), which is a known scavenger of electron holes (Kulmala *et al.*, 1997 ▶). In comparison, crystals of APC5871 show a six times faster rate of radiation-induced specific changes, *R*_R_/Δ*B*_rel_ = 1.8% Å^−2^. Such fast changes in structure factors could in this case result from lattice-destruction phenomena (§[Sec sec3.1.3]3.1.3); however, APC35880 crystals that do not show signs of lattice destruction have a comparable ratio of *R*_R_/Δ*B*_rel_ = 1.6% Å^−2^.

In real space, we expect mainly negative RDDEM peaks, as atoms are being smeared out, but in the case of atoms being shifted positive peaks may also appear at the new positions of the atoms. The typical features of these maps are negative peaks around solvent-exposed carboxyl groups (Borek *et al.*, 2007 ▶; Burmeister, 2000 ▶; Weik *et al.*, 2001 ▶), slowly disappearing Se atoms that, owing to higher atomic number, produce higher peaks, and disappearing S atoms in cysteine residues (Banumathi *et al.*, 2004 ▶; Borek *et al.*, 2007 ▶; Burmeister, 2000 ▶) and in disulfide bridges (Borek *et al.*, 2007 ▶; Banumathi *et al.*, 2004 ▶; Burmeister, 2000 ▶; Weik *et al.*, 2000 ▶; Ravelli & McSweeney, 2000 ▶). Changes around solvent molecules (Borek *et al.*, 2007 ▶) also occur, but they depend strongly on their macromolecular environment. The dominant localized specific changes are sometimes not even at the site of the heavy-atom absorber, as for example in the case of crystals containing nitrates, where, owing to well established radiation chemistry (Saran *et al.*, 1994 ▶), the nitrate anion is a preferred localization site of specific radiation-induced changes compared with heavier atoms such as sulfur (Borek *et al.*, 2007 ▶).

The structures analyzed here show different patterns of radiation-induced specific changes (Table 3 ▶). In the NaI-841 structure the RDDEM shows only nine peaks that are either higher than 5σ or lower than −5σ. Six of these peaks represent negative signal at the site of iodide anions and three of them are signs of decarboxylation of either aspartic or glutamic acid residues (Table 3 ▶). It is interesting to notice that in this structure methionine or cysteine residues do not show signs of specific changes induced by radiation, in contrast to the structure of the same protein not soaked with sodium iodide (data not shown). The small number of specific changes for this structure is consistent with iodide being a known electron scavenger (Kulmala *et al.*, 1997 ▶).

**Figure 2:**
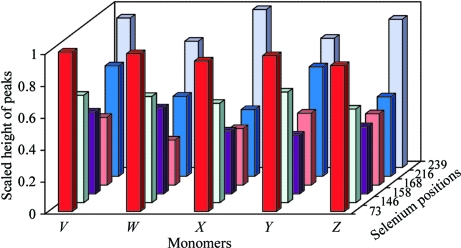
Distribution of height of RDDEM peaks at Se-atom positions for APC35880. The height of the peaks was scaled by dividing the height of each peak by the height of the highest RDDEM peak at a Se atom.

In the case of the crystal structure of SeMet-derivativized APC35880, the largest RDDEM peaks are localized at seleno­methionine residues, at carboxyl moieties and in the solvent area (Table 3 ▶). The APC35880 protein forms an icosahedral assembly with an asymmetric unit of space group *I*23 containing a pentamer. Because of the high symmetry of the atomic environments, the patterns of radiation-induced specific changes are very similar in all subunits of the pentamer (Fig. 2 ▶). The most significant radiation-induced changes are localized at the Se atoms in the SeMet73 and SeMet239 residues. These peaks are three times higher than the peaks localized at SeMet168 and about two times higher than the peaks localized at SeMet158.

**Figure 3:**
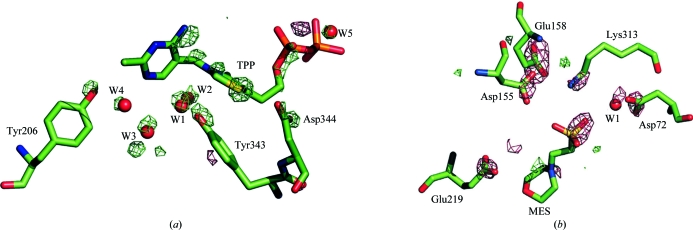
Radiation-induced specific changes around ligand-binding sites in p37n33 (*a*) and TP0655 (*b*). RDDEM contour levels are expressed in root-mean-square units (σ). The green color corresponds to the +5σ level and red to the −5σ level. For clarity, water molecules were relabeled with respect to PDB entries 3e79 and 2v84. For 3e79, W1 = W1035, W2 = W1002, W3 = W1046, W4 = W1028 and W5 = W1090; for 2v84, W1 = W116.

Both the Tp0655 and p37n33 crystal structures show a consistent decarboxylation pattern of glutamic and aspartic acid residues (Table 3 ▶). The most interesting features in both structures are the radiation-induced specific changes localized at bound ligands: on thiamine diphosphate for p37n33 and 2-­(*N*-morpholino)ethanesulfonic acid (MES) for Tp0655 (Fig. 3 ▶). Both ligands contain S atoms, with the peak heights indicating that these S atoms are more sensitive to ionizing radiation than the sulfurs in methionine and cysteine residues. RDDEM features around the thiamine bound in the p37n33 structure show very pronounced effects not only at the atoms of the ligand but also at the binding site (Fig. 3 ▶). This is one of the examples that shows the most pronounced, but not easily chemically interpretable, radiation-induced changes at the active site of the protein.

**Figure 4:**
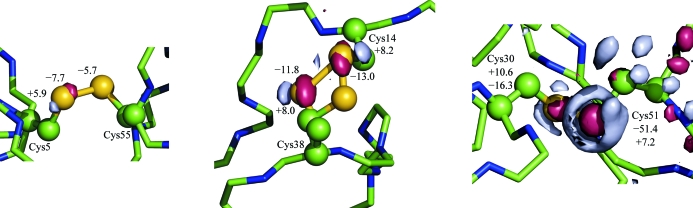
Differences in radiation-induced specific changes at disulfide bridges of BPTI. Red surfaces represent the −5σ level and gray surfaces represent the +5σ level. Cys51 is damaged at a much faster rate than other cysteine residues.

The BPTI crystal structure clearly shows a differential rate of radiation-induced specific damage, as observed previously for lysozyme crystals (Weik *et al.*, 2000 ▶; Banumathi *et al.*, 2004 ▶; Ravelli & McSweeney, 2000 ▶). In the RDDEM map, the most altered disulfide bridge, Cys30–Cys51, has a negative peak of −51.4σ, whereas the other two disulfide bridges show negative peaks of −16.4σ and −7.7σ, indicating that the Cys30–Cys51 disulfide bridge is damaged four to eight times faster than the other two disulfide bridges (Fig. 4 ▶).

All these examples illustrate the incompletely understood complexity of radiation chemistry. What is clearly involved is the migration of excited electronic states (Patten & Gordy, 1960 ▶; Wenger *et al.*, 2005 ▶) and the possibility that atoms dis­placed by one radiation event may return to the starting position owing to another radiation event.

#### 3.1.3. Lattice destruction

Unlike the overall decay and specific changes discussed above, lattice destruction is a sporadic phenomenon; when it occurs, it is highly nonlinear with respect to dose. This behavior indicates the involvement of higher order processes, for example the accumulation of nanoscale gas bubbles (Garrido *et al.*, 2008 ▶; Massover, 2007 ▶; Leapman & Sun, 1995 ▶). Similar to the case of radiation-induced specific changes, the chemistry and physics of lattice destruction are poorly understood.

Lattice destruction manifests itself as a dramatic increase of crystal mosaicity, changes in diffraction spot profiles, a sudden appearance of patterned diffused scattering and changes in diffraction intensities. If such effects appear, the subsequent diffraction data are worthless for structure determination.

**Figure 5:**
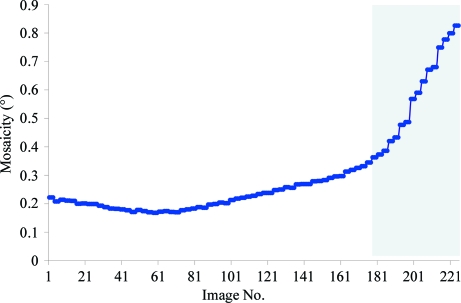
Lattice destruction in an APC5871 crystal. The figure shows a dramatic increase in mosaicity at image 180, suggesting progressive lattice destruction.

The crystal of the Midwest Center for Structural Genomics target APC5871 is a good example of the lattice-destruction phenomenon. Diffraction data for a continuous 220° of oscillation range, with an oscillation step of 0.5°, were acquired for this target and all the diffraction images had a diffraction pattern that extended to high resolution. However, data processing revealed a sudden and fast increase in both mosaicity and relative *B* factor starting at around image 180 (Fig. 5 ▶). Efforts to use all the integrated data did not lead to structure solution. In space group *P*2_1_ the first 180 images were not sufficient to obtain a complete data set. However, using this data set derived only from the well scaling part of diffraction data, which was about 90% complete in terms of unique reflections, resulted in structure solution (data not shown). Considering the nonlinearity of the changes for the total oscillation range and the very low multiplicity of observations for Bijvoet pairs in the data set trimmed to 180 images, the approach of describing the radiation damage by adding parameters for each unique index could not improve the situation in this case. Therefore, where lattice destruction is present, a nonparametric approach in which only part of the data are used seems to be the optimal strategy.

### 3.2. Radiation damage and phasing strategies

In a macromolecular crystallographic experiment, crystal destruction, arising either from lattice disorder or radiation-induced overall decay, cannot be overcome. For an average experiment, this effective limit is reached when the scaling *B* factor (*B*_rel_) reaches the range 10–40 Å^2^. Traditional diffraction data-processing analysis ignores radiation-induced specific changes, generating negative quality-assessment statistics when such changes are comparable to or larger than other measurement errors. In consequence, frequently the strategy is to minimize the exposure and, as a byproduct, to also limit the overall decay by collecting a much smaller number of diffracted photons than physics allows. However, even when complete coverage of reciprocal space is achieved, *i.e.* a complete data set is acquired, it is still worthwhile con­tinuing data collection to improve the overall multiplicity of observations and counting statistics (Dauter *et al.*, 1999 ▶). Owing to the overall decay of diffraction intensities, the gain from additional data decreases over the time of data collection. However, as the overall decay is fully corrected in the data analysis and the data are properly weighted (§[Sec sec3.1.1]3.1.1; Otwinowski *et al.*, 2003 ▶), these weaker observations improve the overall data quality even if statistics such as *R*_merge_, *R*_sym_, *R*_p.i.m._ and *R*_r.i.m._ (reviewed in Weiss, 2001 ▶) unweighted for radiation decay may be pointing the other way. The impact of both corrected and uncorrected specific radiation-induced changes is more complex than that of the overall decay and how we should approach these changes depends on the choice of phasing strategy.

#### 3.2.1. Experimental phasing *versus* direct methods

Molecular and isomorphous replacement as well as phase-extension methods are phasing techniques that rely on native intensities only. Even uncorrected specific radiation-induced changes that are large in comparison to the measurement error still represent a relatively small fraction of native intensities, so they essentially have no influence on our ability to solve structures using direct methods. To a good approximation, a data set uncorrected for radiation-induced specific changes represents a structure in the middle of data collection (Ramagopal *et al.*, 2005 ▶; Zwart *et al.*, 2004 ▶; Borek *et al.*, 2007 ▶), which is somewhat altered in terms of the occupancy of particular atoms but not significantly rearranged. In terms of the difficulty of solving the structure using native intensities, there is no difference between a pristine structure and the structure in the middle of data collection. If the radiation-induced specific changes are ignored in the data analysis, the refined model represents the structure that has already been irradiated. In many cases, even with high radiation doses, modeling of such a dose-averaged state will not distort biological interpretations. However, if the structural model is used to answer questions involving, for example, changes of redox state, light (radiation) sensitive centers or heavy-atom clusters, then caution should be exercised when interpreting such structural models, regardless of the approach used for phasing. In such cases a parametric approach involving extrapolating intensities to zero dose may be highly beneficial for biological and chemical interpretations and data-collection strategies should be adjusted accordingly to optimize the use of a parametric approach.

In the case of the SAD and MAD types of experimental phasing, the phasing signal is defined by relatively small differences that could easily be smaller than radiation-induced specific changes over the lifetime of the crystal (Table 2 ▶; Borek *et al.*, 2003 ▶; Bourenkov & Popov, 2006 ▶). Additionally, in specific cases the heavy-atom scatterers used for phasing may undergo fast specific changes (Ramagopal *et al.*, 2005 ▶; Ennifar *et al.*, 2002 ▶; Holton, 2007 ▶). These situations create both problems and opportunities. When using a parametric approach to obtain zero-dose intensities, the main problem arises from the need to simultaneously determine a number of parameters for each unique *hkl* index: the zero-dose extrapolated intensity *I*_d0_, the Bijvoet difference, the native intensity linear rate of change with respect to dose and potentially other parameters, *e.g.* the Bijvoet difference rate of change or the native intensity quadratic and higher order dependence on dose. For typical experiments, the level of measurement error is comparable to the magnitude of all the parameters other than *I*_d0_, so multiparameter determination of their values with a limited number of observations is potentially unstable unless special care is taken, for example by using Tikhonov regularization (Tikhonov & Arsenin, 1977 ▶). After these parameters have been determined, the opportunity arises to use them as an additional phasing source if a model of specific changes can be generated. One of the possible examples is the case of a heavy-atom scatterer that diffuses far enough to be considered as an atom that disappears during exposure and with an occupancy modeled in a dose-dependent (or time-dependent) fashion (Schiltz & Bricogne, 2007 ▶; Schiltz *et al.*, 2004 ▶). In practice, this situation is likely to happen for mercury covalently bound to sulfur in cysteine (Ramagopal *et al.*, 2005 ▶) and for halogen atoms in derivatives of nucleotides (Ennifar *et al.*, 2002 ▶). In typical cases the situation is more complicated, as the majority of changes may be scattered over a large number of places (Borek *et al.*, 2007 ▶). Also, since other heavy atoms diffuse by short distances, they would have to be modeled as changing shape during the experiment rather than as disappearing. However, even a very rough approximation of atoms disappearing rather than changing shape may sometimes work if high-resolution data are available; so, for instance, the BPTI case (Fig. 3 ▶) can be solved from radiation-induced changes at S atoms (data not shown).

Specific changes can be considered as non-isomorphism induced by X-rays during data collection. However, there are potentially other sources of non-isomorphism that may interfere with phasing and with estimation of the magnitude of specific radiation-induced changes.

#### 3.2.2. Sources of non-isomorphism in data

There are four main sources of usually undesired non-isomorphism in data: (i) rotational pseudosymmetries which are too weak to be considered a lower symmetry case; (ii) variability of the crystal lattice periodicity (unit-cell parameters) within the crystal, for example induced by a variable rate of cooling during cryo-preservation; (iii) variability between crystals, either spontaneous or induced by soaking; and (iv) effects within crystals induced by X-ray radiation during data collection.

When analyzing specific changes induced by radiation, it is important to consider all other possible sources of non-isomorphism because they may be a more significant source of the problem than radiation damage itself. Additionally, when analyzing the differences between symmetry-equivalent measurements, we need to consider whether they arise from dose-dependent effects or potentially from the other above-mentioned sources.

#### 3.2.3. The best crystal *versus* many crystals *versus* the only crystal

Nowadays, data from one crystal are typically used for phasing. This strategy relies on choosing the best sample, for which the most critical characteristics are (i) the size of the sample, as it affects the diffraction power; (ii) microscopic order, which should be the same for various samples of the same type, but often, owing to variability in cryoprotection, is not; (iii) macroscopic order, with the sample preferably being a single crystal with a mosaicity small enough to avoid spot overlaps; (iv) the types of non-isomorphism discussed in §[Sec sec3.2.2]3.2.2 and (v) a special case of macroscopic disorder, *i.e.* merohedral twinning.

For particular projects, these characteristics may often be correlated; for example, weak macroscopic order very often correlates with non-isomorphism within the sample. The first three characteristics are immediately visible in the data, but the last two are only consequential during data merging or phasing, with non-isomorphism often not being identified as such. Non-isomorphism within the sample is often visible even in the benchmark crystals, *e.g.* thaumatin or tetragonal lysozyme. The choice of the best crystal should be made based on minimizing all five types of problems mentioned above. However, owing to merohedral twinning and non-isomorphism usually becoming apparent after data collection, the crystals are often selected based only on the first three characteristics, even if sometimes it is better to sacrifice these three to compensate for the impact of the last two.

The crystal size and the quality of its microscopic and macroscopic orders correlate well with the values of various types of *R*_merge_ and *R*_sym_ indicators; for this reason, they have frequently been used to select the best data set. However, when applied to data with radiation damage these indicators are quite misleading with respect to the optimal data-collection strategy, prompting experimenters to use less-than-optimal levels of exposure. In most cases, even without correcting for radiation-induced specific changes, more data or data with higher exposure would be advantageous. When using corrective procedures, the total exposure limit is determined by the lifetime of the sample, which is typically in the range 10–40 MGy, and one should take advantage of it whenever possible. In terms of aquiring more information, the point of diminishing returns depends on the initial diffraction resolution limit. The rule of thumb is to limit the data to the range where the *B*_rel_ factor increases by two to four times the inital resolution limit squared, *e.g.* for 3.0 Å data it would be a *B*_rel_ increase of 18–36 Å^2^, whereas for 1.0 Å data it would be a *B*_rel_ increase of 2–4 Å^2^.

An alternative to the best crystal strategy is to average the data collected from many crystals. This was the original strategy for macromolecules and works well when crystals are isomorphous. For crystals that cannot be cryocooled, *e.g.* some viruses, or for crystals that have too little diffracting power to survive the collection of a complete data set, it may be the only viable strategy. Multi-crystal data averaging works much better with molecular-replacement and phase-extension techniques, which use only native intensities. As the main limitation in using many crystals is the non-isomorphism between them, experimental phasing with many crystals is particularly challenging. As the phasing differences in SAD and MAD are typically small, constituting 2–5% of the native signal, if multiple crystals have to be used for *de novo* structure determination, SIR and MIR methods have the advantage of generating larger phasing differences, typically 10–30% of the native signal. To efficiently use multiple crystals for phasing it is beneficial to identify the sources of non-isomorphism, *e.g.* do they result from variability in cryocooling stabilization conditions? Are they induced by soaking with particular concentrations of heavy-atom compound? Are the crystals inherently polymorphic?

Sometimes only one promising crystal that is of sufficient quality for complete data collection is available. In such a situation, the best strategy is to collect a complete data set with minimal exposure, followed by collecting data with higher exposure. In the first data-collection round, the strongest reflections are measured and represent the state of the relatively undamaged structure. As these reflections have already been precisely measured, we do not have to worry about them being saturated at higher exposure levels. Additionally, merging statistics from this first data set can predict the decay rate, phasing signal magnitude and potential non-isomorphisms, allowing adjustment of the data-collection strategy.

#### 3.2.4. Minimizing or maximizing the multiplicity of observations as a planning goal

There are two main decisions to be made when planning an X-ray experiment. Firstly, adjusting the total dose *versus* the extent of acceptable radiation damage and, secondly, splitting the total dose between individual exposures. Addressing these two questions separately is the best way to arrive at the dose-per-exposure decision. Increasing the multiplicity of observations, *i.e.* extending the total oscillation range, averages out many systematic errors that are differently affected in symmetrically equivalent reflections. On the other hand, adding more read-outs from energy-integrating detectors, *e.g.* charge-coupled devices (CCDs) or image plates, slightly increases the random-error component of merged intensity. The decision about how many oscillations to collect is often affected by other factors, *e.g.* detector read-out time, problems with handling large volumes of data *etc.* These types of limitations are diminishing very dynamically and their impact needs to be re-evaluated. Historically, in many diffraction experiments the strategy of data collection was adjusted to collect a complete data set with minimal total oscillation range, which is equivalent to minimizing the multiplicity of observations. Now it is clear that the gain from increasing the multiplicity of observations while keeping the total dose constant is significant (Dauter *et al.*, 1999 ▶) and modern data-collection strategies should accommodate this observation.

When determining specific radiation changes and potentially other high-order terms, a high multiplicity of observations (at least eightfold) is particularly valuable. As the technology evolves, the historical approach of minimizing the multiplicity of observations should be re-evaluated and con­sideration should be given to replacing it with lower levels of controlled exposure, generating higher multiplicity of observations.

## 4. Summary

The last few years have brought strong X-ray sources, fast detectors, vast computational speed and storage to macromolecular crystallography, removing previous limitations on data collection and facilitating the determination of more challenging crystallographic structures. This has brought into focus new limitations, in particular those resulting from radiation damage; consequently, the need to address them or even to exploit them has arisen. Our article discusses this new environment in which the analysis of specific radiation damage begins to be considered; it is bound to become a major com­ponent of future data analysis. The experimental approach will evolve accordingly; in particular, a high multiplicity of observations will be acquired, observed structure factors will be extrapolated to zero dose, heavy-atom-based phasing signals will be corrected for specific radiation-induced changes and possibly specific radiation damage will become more widely used as a phasing component. Instead of radiation damage being considered as diffraction-pattern decay and accordingly corrected, it will be fully analyzed in all its complexity, enabling the determination of large macromolecular structures with improved accuracy.

## Figures and Tables

**Table 1:** Data-collection and processing statistics Values in parentheses are for the last resolution shell.

	APC5871	APC35880	BPTI	NaI-841	p37n33	Tp0655	VAV
Wavelength (Å)	0.97940	0.97934	0.9090	1.7280	1.54178	1.54178	0.97874
Beamline	19-ID	19-ID	X11	19-BM	R-AXIS IV/F-RE	R-AXIS IV/F-RE	19-ID
Oscillation range per run (°)	220†	180	90 each	180	400	636.9	240 each
Oscillation step (°)	0.5	0.3	1st run, 0.5; 2nd run, 1.0; 3rd run, 2.0; 4th run, 3.0	0.6	0.5	0.3	1st run, 0.5; 2nd run, 0.5; 3rd run, 0.5
No. of runs	1	1	4	1	1	1	3
Space group	*P*2_1_	*I*23	*P*4_3_2_1_2	*P*4_1_2_1_2	*P*2_1_2_1_2_1_	*C*222_1_	*P*2_1_
Unit-cell parameters							
*a* (Å)	146.4	210.1	51.9	118.1	67.2	172.9	85.0
*b* (Å)	83.1	210.1	51.9	118.1	124.3	179.2	58.8
*c* (Å)	146.9	210.1	43.0	124.1	53.2	51.8	161.1
α (°)	90.0	90.0	90.0	90.0	90.0	90.0	90.0
β (°)	90.8	90.0	90.0	90.0	90.0	90.0	97.3
γ (°)	90.0	90.0	90.0	90.0	90.0	90.0	90.0
Resolution (Å)	50.00–1.93	50.00–2.68	20.00–0.87	50.00–2.95	50.00–1.55	20.00–1.95	50.00–2.70
Last resolution shell (Å)	1.95–1.93	2.70–2.68	0.89–0.87	3.06–2.95	1.56–1.55	1.97–1.95	2.72–2.70
No. of unique reflections	242086	42946	46278	19110	61950	48357	42185
Completeness (%)	91.4 (40.4)	100.0 (99.9)	95.0 (88.8)	99.0 (99.5)	94.2 (88.2)	81.9 (2.5)	96.9 (71.5)
*R*_merge_‡ (%)	4.1 (27.7)	11.1 (70.6)	2.4 (49.6)	10.8 (77.1)	3.0 (42.7)	3.1 (46.4)	8.0 (89.2)
〈*I*/σ(*I*)〉§	14.0 (1.9)	33.9 (4.5)	101.9 (3.4)	18.9 (2.1)	102.0 (6.5)	78.6 (1.9)	30.8 (1.9)
Multiplicity of observation (overall/anomalous)	2.00/1.2	21.6/11.1	8.7/4.6	8.6/4.6	15.3/8.0	20.2/10.7	14.1/7.3

† Data-processing statistics represent only a 90° range of oscillation, since this was the oscillation range used for structure solution as discussed in §[Sec sec3.1.3]3.1.3.

‡ After correcting for radiation-induced specific changes.

§ The 〈*I*/σ(*I*)〉 value is calculated based on merged symmetrically equivalent observations.

**Table 2:** Statistical indicators of global and specific radiation-induced changes Δ*B* represents the scaling (relative) *B*-factor increase, which is proportional to the dose; *R*_R_ describes the magnitude of radiation-induced specific changes after correction for the overall decay. The ratio *R*_R_/Δ*B* compares the rate of radiation-induced specific changes accumulated between different crystals and represents the fraction of structure-factor change per unit of scaling *B*-factor increase. Δ_ano_ represents the level of the anomalous signal obtained from diffraction data after applying corrections for overall and specific radiation-damage effects as well as various scaling effects (Borek *et al.*, 2003 ▶; Otwinowski *et al.*, 2003 ▶).

	APC5871	APC35880	BPTI	NaI-841	p37n33	Tp0655	VAV
Δ*B* (Å^2^)	2.4	5.4	2.4	20.8	5.4	6.3	9.9
*R*_R_ (%)	4.4	8.6	2.2	6.7	7.1	5.9	3.6
*R*_R_/Δ*B* (% Å^−2^)	1.8	1.6	0.9	0.32	1.3	0.93	0.36
Δ_ano_ (%)	6.98	4.21	0.67	9.66	0.65	0.75	4.09

**Table 3:** Summary of the 15 highest peaks observed in a radiation-damage difference map The APC5871 crystal was not used in this analysis because the very low multiplicity of observations did not allow zero-dose extrapolation, which is necessary for RDDEM coefficient determination of the map. The VAV protein was excluded, as the final model has not yet been deposited. For the p37n33 structure, determined by sulfur SAD phasing (Dann, unpublished work), PDB entry 3e79 (Sippel *et al.*, 2008 ▶) was used as a reference point. For each protein, all peaks with σ values outside the ±5σ (r.m.s.) range were analyzed. Numbering of solvent molecules follows that of the PDB depositions; however, in the case of the iodide soak, for which only the native structure has been deposited, the water-molecule number is specified to provide the location of the I-atom positions. Superscripts indicate the protein chain, whereas subscripts indicate the type of atom when the RDDEM peak could be assigned to a single atom.

Protein	APC35880	NaI-841	p37n33	Tp0655
PDB code	1t0t	1r61	3e79	2v84
1	M_SE_73^*V*^ (−10.86)	H_2_O2015^*A*^ and H_2_O2016^*A*^ (−8.02) iodide	Thiamine_S1_ (−13.34)	E150^*A*^ (−11.24) decarboxylation
2	M_SE_73^*W*^ (−10.74)	H_2_O2023^*B*^ and H_2_O2024^B^ (−6.16) iodide	E146^*A*^ (−8.89) decarboxylation	MES_S_ 1326 (−9.23)
3	M_SE_239^*X*^ (−10.65)	H_2_O2025^*A*^ (−6.09) iodide	E146^*A*^ (−8.86) decarboxylation	E211^*A*^ (−7.16) decarboxylation
4	M_SE_239^*V*^ (−10.19)	H_2_O2061^*A*^ (−5.58) iodide	E79^*A*^ (−8.31) decarboxylation	M_SD_152^*A*^ (+6.79) position change
5	M_SE_73^*X*^ (−10.13)	H_2_O2012^*A*^ (−5.54) iodide	K403^*A*^ (−8.15) decarboxylation	D147^*A*^ (−6.41) decarboxylation
6	M_SE_73^*Y*^ (−9.90)	E124^*A*^ (−5.51) decarboxylation	E393^*A*^ (−7.94) decarboxylation	D64^*A*^ (−6.24) decarboxylation
7	M_SE_239^*Z*^ (−9.22)	H_2_O2014^*A*^ (−5.25) iodide	E157^*A*^ (−7.83) decarboxylation	Solvent area (−6.16)
8	M_SE_73^*Z*^ (−9.05)	D141^*B*^ (−5.20) decarboxylation	N_O_213^*A*^ (−7.76)	M_SD_145^*A*^ (+6.16) position change
9	M_SE_239^*W*^ (−8.61)	D70^*A*^ (−5.18) decarboxylation	H_2_O1035^*A*^ (−7.72)	M_SD_145^*A*^ (−6.10) position change
10	M_SE_239^*Y*^ (−8.22)	Solvent area (4.99) possible iodide	H_2_O1092^*A*^ (−7.68)	E263^*A*^ (−5.94) decarboxylation
11	D16^*V*^ (−7.86) decarboxylation	Solvent area (−4.91) possible iodide	Thiamine_N4_ (−7.67)	G_O_115^*A*^ (+5.93)
12	M_SE_216^*V*^ (−7.53)	D70^*A*^ (−4.91) decarboxylation	E393^*A*^ (−7.65) decarboxylation	H_2_O2128^*A*^ (−5.93)
13	M_SE_146^*V*^ (−7.31)	D_N_141^*B*^ (+4.83)	E393^*A*^ (−7.49) decarboxylation	K305^*A*^ (−5.86) position change
14	M_SE_146^*W*^ (−7.26)	P_CG_195^*A*^ (+4.8)	D388^*A*^ (−7.48) decarboxylation	E104^*A*^ (−5.80) decarboxylation
15	D147^*Z*^ (−7.18) decarboxylation	M_SD_181^*B*^ (−4.74)	D159^*A*^ (−7.44) decarboxylation	K305^*A*^ (−5.68) position change
No. of peaks†	57	9	104	29

† No. of peaks that are either lower than −5σ or higher than 5σ.
